# The Signal Transduction Protein PII Controls the Levels of the Cyanobacterial Protein PipX

**DOI:** 10.3390/microorganisms11102379

**Published:** 2023-09-23

**Authors:** Antonio Llop, Lorena Tremiño, Raquel Cantos, Asunción Contreras

**Affiliations:** Departamento de Fisiología, Genética y Microbiología, Universidad de Alicante, 03690 San Vicente del Raspeig, Spain; antonio.llop@ua.es (A.L.); lorena.tremino@ua.es (L.T.); raquel.cantos@ua.es (R.C.)

**Keywords:** NtcA, *Synechococcus elongatus*, nitrogen regulation network, light and dark conditions, PipX toxicity, protein interaction, energy sensing, mutational analysis

## Abstract

Cyanobacteria, microorganisms performing oxygenic photosynthesis, must adapt their metabolic processes to environmental challenges such as day and night changes. PipX, a unique regulatory protein from cyanobacteria, provides a mechanistic link between the signalling protein PII, a widely conserved (in bacteria and plants) transducer of carbon/nitrogen/energy richness, and the transcriptional regulator NtcA, which controls a large regulon involved in nitrogen assimilation. PipX is also involved in translational regulation through interaction with the ribosome-assembly GTPase EngA. However, increases in the PipX/PII ratio are toxic, presumably due to the abnormally increased binding of PipX to other partner(s). Here, we present mutational and structural analyses of reported PipX-PII and PipX-NtcA complexes, leading to the identification of single amino acid changes that decrease or abolish PipX toxicity. Notably, 4 out of 11 mutations decreasing toxicity did not decrease PipX levels, suggesting that the targeted residues (F12, D23, L36, and R54) provide toxicity determinants. In addition, one of those four mutations (D23A) argued against the over-activation of NtcA as the cause of PipX toxicity. Most mutations at residues contacting PII decreased PipX levels, indicating that PipX stability would depend on its ability to bind to PII, a conclusion supported by the light-induced decrease of PipX levels in *Synechococcus elongatus* PCC7942 (hereafter *S. elongatus*).

## 1. Introduction

Cyanobacteria, phototrophic prokaryotes that perform oxygenic photosynthesis, are the main contributors to marine primary production [1,2] and have a very important ecological impact on global carbon, nitrogen, and oxygen cycles. They have evolved sophisticated systems to maintain the homeostasis of carbon/nitrogen assimilation (reviewed by [3,4]), the two most abundant elements in all living forms. Cyanobacteria can use different nitrogen sources that are first converted into ammonium and then incorporated into amino acids and other N-containing compounds via the glutamine synthetase-glutamate synthase (GS-GOGAT) pathway using 2-oxoglutarate (2-OG) as a carbon skeleton [5]. The metabolite 2-OG, a universal indicator of the intracellular carbon-to-nitrogen balance [6,7], appears to be particularly suitable for this role in cyanobacteria [4].

In bacteria and plants, 2-OG is sensed by PII, a widely distributed and highly conserved signal transduction protein. PII regulates the activity of proteins involved in nitrogen and carbon metabolism by direct protein-protein interactions [8], perceiving metabolic information through the competitive binding of ATP or ADP and the synergistic binding of ATP and 2-OG [9,10].

**Figure 1:**
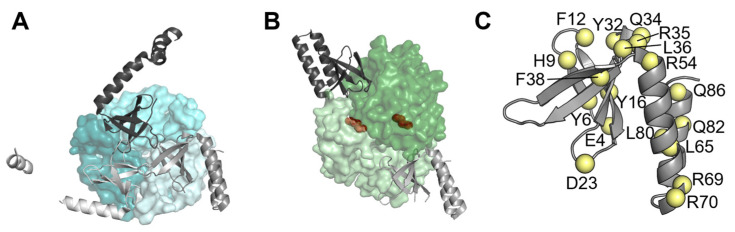
PipX in complex with PII or NtcA, or illustrating the position of residues discussed in this work. (**A**) PipX-PII (PDB:2XG8), (**B**) PipX-NtcA (PDB:2XKO), and (**C**) PipX (chain E of PDB file 2XG8). PipX structures are shown in ribbon representations and different hues of grey. PII and NtcA are shown in surface representation, with each subunit in a different hue of blue (PII) or green (NtcA). In (**B**), NtcA-bound 2-OG molecules are shown in spheres in brown. In (**C**), yellow spheres indicate the location of the indicated residues in the PipX structure.

The first PII targets identified in cyanobacteria were detected in *Synechococcus elongatus* PCC7942: NAGK (N-Acetyl Glutamate Kinase) and PipX (PII interacting protein X), a small protein of 89 amino acids restricted to cyanobacteria [11,12,13,14,15,16]. The N-terminal domain of PipX (residues 1–53) is a Tudor-like domain [17] that is assimilated to the KOW domain [18]. The C-terminal domain (residues 54–89) is composed of two alpha-helices. In the PipX-PII complexes (Figure 1A), one PII trimer sequesters three PipX molecules [17]. PipX-PII complexes from *Anabaena* sp. PCC 7120 are virtually identical to those of *S. elongatus* [19].

Cyanobacterial genomes always contain at least as many copies of the essential gene *glnB* (encoding PII) as of the non-essential gene *pipX* [20]. In addition, loss-of-function mutations at *pipX* allow inactivation of the *glnB* gene in *S. elongatus* [21,22,23,24,25], suggesting that a relatively high PII/PipX ratio is needed to sequester PipX to prevent “PipX toxicity," caused by uncontrolled binding to additional partners.

PipX also binds to the global transcriptional regulator NtcA, which is involved in nitrogen assimilation in cyanobacteria [12,26,27]. PipX coactivates the NtcA regulon in response to nitrogen limitation of [28,29]. The PipX–NtcA complex (Figure 1B; [17]) consists of one active (2-OG-bound) NtcA dimer and two PipX molecules. Each NtcA subunit binds one PipX molecule. PipX stabilises the conformation of NtcA, which is transcriptionally active, and probably helps local recruitment of RNA polymerase. The binding of PipX to PII or NtcA is antagonistically tuned by 2-OG levels; whereas high levels of 2-OG favour the interaction of PipX with NtcA, they prevent the PipX-PII interaction [12,17,22,28].

PipX uses the same surface as its TLD/KOW domain to bind to either 2-OG-bound NtcA, stimulating DNA binding and transcriptional activity, or to 2-OG-free PII. PII sequestration of PipX at low 2-OG renders PipX unavailable for NtcA binding and activation, reducing the expression of NtcA-dependent gene targets [16,17,19,21,22,23,30]. In addition, the interaction between PII and PipX is highly sensitive to fluctuations in the ATP/ADP ratio, and thus the energy state of the cells [31,32].

In *S. elongatus, pipX* forms a bicistronic operon with the downstream gene *pipY* [33], encoding a member of the widely distributed and highly conserved family of pyridoxal phosphate (PLP)-binding proteins (COG0325/PLPBP) that are involved in vitamin B_6_ and amino acid homeostasis [34,35]. Previous studies supported functional interactions between PipX and PipY in cyanobacteria [33,36,37].

While the identification of PipX as a binding partner for PII or NtcA led to the characterisation of PipX as a transcriptional player in the context of carbon/nitrogen metabolism, more recent studies suggest the influence of PipX in additional processes and at additional regulatory levels. Yeast three-hybrid searches with PipX–PII as bait identified the transcriptional regulator PlmA, a protein found exclusively in cyanobacteria, as an additional PipX target [13]. Transcriptomic analyses using *pipX* mutant derivatives revealed a strong connection between PipX and translation [28]. Gradient profiling by sequencing (Grad-seq) showed that PipX co-localizes with either metabolic regulators PII, NtcA, and PlmA or with RNA-protein complexes involved in transcription, RNA metabolism, and translation initiation [38]. Last but not least, cyanobacterial synteny [14] led to the identification of the ribosome-assembly GTPase EngA as a PipX regulatory target [39,40], thus providing additional evidence of the regulatory connections between PipX and ribosome function in *S. elongatus*.

The aim of this work was to gain insights into the determinants involved in the toxic interaction of PipX with its partner(s) by expanding previous phenotypic analyses in *S. elongatus*, where we studied the effect of point mutations at *pipX* on toxicity phenotypes and/or on PipX levels. PipX residues that were mutated in the present or previous studies are shown in Figure 1C. Here we found that most of the mutations decreasing toxicity also decreased protein levels. Since most of these mutations target residues involved in binding to PII or NtcA, the results further suggest that the in vivo stability of PipX is affected by its ability to bind to PII. In addition, the four single-point mutations that did not impair protein levels despite decreasing PipX toxicity identify residues that appear to be directly involved in toxicity. Last but not least, we show that disruption of PipX-PII complexes in *S. elongatus* cultures under environmentally relevant conditions, such as the transition from darkness to light, transiently decreases PipX levels.

## 2. Materials and Methods

### 2.1. Cyanobacterial Growth Conditions

*S. elongatus* cultures were routinely grown at 30 °C in BG11 media (BG11_0_ plus 17.5 mM sodium nitrate (NaNO_3_) and 10 mM HEPES/NaOH pH 7.8; [41]), under constant illumination provided by cool white-fluorescent lights in baffled flasks (shaking: 150 rpm, 70 μmol photons m^−2^s^−1^) or on plates (50 μmol photons m^−2^s^−1^). For solid media, 1.5% (*w*/*v*) agar and 0.5 mM sodium thiosulfate (Na_2_S_2_O_3_; after autoclaving) were added. Transformations were performed essentially as described [42]. To select genetically modified strains, solid media were supplemented with the antibiotics chloramphenicol (Cm; 3.5 μg mL^−1^), streptomycin (Sm; 15 μg mL^−1^), or kanamycin (Km; 12 μg mL^−1^).

To initiate liquid cultures, strains were inoculated into 30 mL of BG11 and incubated under standard conditions for two days. Subsequently, culture stocks were adjusted to an initial optical density (OD_750nm_) of 0.1 and grown until they reached 0.5–0.7 in 25–30 mL. Optical density was measured in an Ultrospec 2100 pro UV–Vis Spectrophotometer (Amersham, Buckinghamshire, UK). For light transition experiments, cultures at an OD_750nm_ of 0.7 were subjected to 12 h of darkness before being transferred to light.

### 2.2. Plasmid and Strains Construction

Strains and plasmids used in this work are listed in Table 1 and Table 2, respectively, and oligonucleotides in Table S1. Cloning procedures were carried out in *Escherichia coli* DH5α using standard techniques [43]. All constructs were analysed by automated dideoxy DNA sequencing.

Plasmids pUAGC948, pUAGC945, pUAGC937, and pUAGC618 were obtained by QuickChange mutagenesis using pUAGC410 as a template and, respectively, PipX-H9A-1F/1R, PipX-Y16A-1F/1R, PipX-R70A-1F/1R, and PipX-L80Q-1F/1R primer pairs.

*S. elongatus* C.K1X*Y derivatives were generated by the transformation of the corresponding plasmids into ∆*pipX*. Strain verification and segregation analysis of the Km^R^ clones were carried out with the primers PipX-126F and PipX-5R.

To check segregation of the null allele *glnB*::CS3, plasmid pUAGC701 was transformed into CK1X^H9A^Y, CK1X^Y16A^Y, CK1X^R70A^Y, and CK1X^L80Q^Y strains, and clones were subsequently analysed with primers Glnb-1F and Glnb-1R.

### 2.3. Protein Extraction and Immunodetection Assays

10 mL of cultures were sampled at different times and quickly harvested by 6 min centrifugation at 7300× *g* (4 °C), flash frozen in liquid nitrogen, and stored at 20 °C until use. The pellets were resuspended in 60 μL of lysis buffer (25 mM Tris/HCl pH 7.5, 0.4 mM EDTA, 1 mM DTT, 0.8 mg/mL protease inhibitor, 50 mM NaCl), and cells were disrupted using a spoon of 0.1 µm glass beads, as described in [13]. Mixtures were subjected to three cycles of 60 s at a speed of 5 m/s in a high-speed homogenizer Minibeadbeater, followed by 60 s at 4 °C after each cycle. Samples were centrifuged (5500× *g* for 5 min), and the supernatant fractions (crude protein extracts) were transferred to a new tube and stored at −20 °C until needed.

Protein concentrations were estimated by the Bradford method using the Pierce^TM^ detergent-compatible Bradford assay kit (ThermoScientific, Waltham, MA, USA) in a VICTOR3^TM^ 1420 Multilabel Plate Reader (PerkinElmer, Waltham, MA, USA). For immunodetection, 60 µg of total protein extract was loaded into a sodium dodecyl sulphate polyacrylamide gel (SDS-PAGE; 15% polyacrylamide). The gel electrophoresis was followed by immunoblotting onto 0.1 μm polyvinylidene fluoride membranes (from GE Healthcare), and the membranes were subsequently blocked with Tris-Buffered Saline (TBS; 20 mM Tris/HCl pH 7.5, 500 mM NaCl) solution containing 5% non-fat dried milk for 30 min at room temperature and then incubated overnight in TBS with 2% non-fat dried milk and the primary antibody. Membranes were then incubated for 1.5 h at room temperature with a 1:150,000 dilution of ECL rabbit IgG, HRP-linked F(ab’)2 fragment (from a donkey; GE Healthcare). The signal was detected with SuperSignal WestFemto reagent (ThermoScientific) in a Biorad ChemiDoc Imager using the automatic exposure mode and avoiding pixel saturation or using X-rays and scanning the films. All the membranes were immunodetected first with a 1:5000 dilution of primary anti-PipX antibody and then with a 1:5000 anti-PlmA antibody. At least two independent western-blot assays of one to three independent clones of each strain were performed for each of the mutant strains. Antisera against PipX (Pineda Antikörper Service, Berlin, Germany) and PlmA (Genosphere Biotechnologies, Paris, France) were produced in rabbits.

### 2.4. Computational Methods

Graphical representations of the protein structures were generated with PyMOL (The PyMOL Molecular Graphics System, Version 1.7.1.7 Schrödinger, LLC). Atom-atom contacts were automatically calculated using the default range defined in LigPlot^+^ Version v.2.2.8 [45].

Protein intensity levels were quantified from the Western blot images using the ImageJ software version 1.53K. Bands were picked up using the “rectangle” function, and the area plot corresponding to the intensity was measured with the “wand” tool. Each area from the PipX immunodetection was normalised using the corresponding area of PlmA and referred to the control strain. Statistical analysis of the results was performed in the RStudio [46] programme. Detailed quantifications of protein expressions can be found in Table S2.

## 3. Results and Discussion

### 3.1. PipX Toxicity Is Altered by Point Mutations Targeting Residues at Each of Its Two Domains

**Figure 2:**
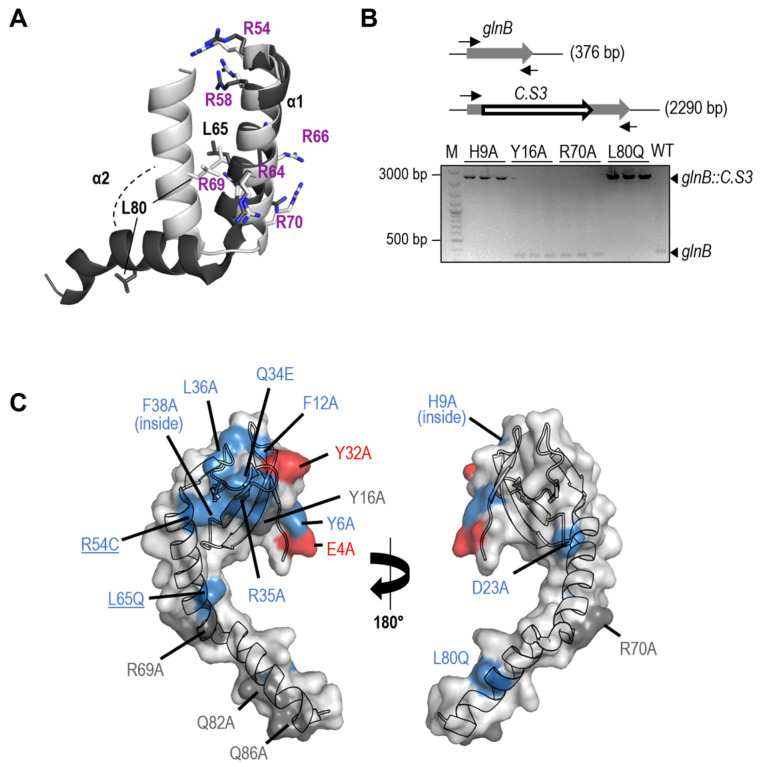
PipX point mutations and toxicity. (**A**) Ribbon representation of C-terminal helices of PipX in “flexed” (light grey; taken from chain D of 2XKO) and “extended” (dark grey, taken from chain D of 2XG8) forms. The discussed residues are indicated, with arginines in purple. Selected side chains are shown (stick representation), with N atoms coloured blue. (**B**) Schematic representation of the wild-type (*glnB)* and mutant (*glnB::C.S3) glnB* alleles whose segregation in CK1X*Y strains is shown below by PCR analysis. The positions of primers and expected sizes of PCR products are indicated. (**C**) Two views of the PipX subunit structure (chain D of PDB file 2XG8) in the extended conformation, with the surface represented in semi-transparent form, rendering visible the fold of the chain in cartoon representation. Underlined residues indicate spontaneous mutations. The location and phenotype conferred by each mutation are illustrated with the following colour code: grey, neutral; blue, loss-of-function; red, gain-of-function.

The identification, while trying to inactivate *glnB* in *S. elongatus*, of spontaneous suppressor mutations at the promoter or at coding sequences of the *pipX* gene [22] indicated that decreasing PipX levels and/or activity makes PII dispensable. However, the mechanism involved in PipX toxicity remains to be addressed. Among the spontaneous suppressor mutations obtained in the different studies, only two were single substitutions (R54C and L65Q) and could thus give clues on PipX residues that may play a role in toxicity in the absence of PII. Their location at the C-terminal domain of PipX (Figure 1 and Figure 2A), which is not involved in the well-characterised interactions with PII and NtcA partners, suggests a rather important role for this domain in PipX toxicity.

R54 is the first of the six arginine residues of a basic R-rich patch located at alpha helix 1. Although R54 only makes one direct and one water-mediated hydrogen bond with NtcA in the available structures, the R54C substitution impaired Y2H interactions with NtcA [17,22]. L65 interacts with L80 of alpha helix 2 in the “flexed” conformation of the two-helix C-terminal domain of PipX and both residues are exposed in the “extended” conformation. The flexed form is found in all PipX-NtcA structures, in some of the PipX-PII molecules [17], and when PipX is not bound to its partners [47]. While L65Q did not impair Y2H interactions with PII or NtcA, it disrupted three-hybrid interactions of PipX-PII with PlmA [13].

Like L65Q, and in contrast to R54C, the L80Q substitution also disrupted the three-hybrid interactions of PipX-PII with PlmA, supporting the involvement of L65 and L80 in common functions.

The role of residues L65 and L80 in promoting conformational changes in PipX prompted us to examine the toxicity of the L80Q variant. Along similar lines, we also examined the toxicity of the R70A variant. R70, located in the R-rich patch of the C-terminal domain, is one of the PipX residues with the highest mobility, and it has been proposed to act as a hinge for the opening of the second helix [47]. The distinction between neutral and loss-of-function mutations for PipX toxicity is carried out by inactivation of the *glnB* gene in a CK1XY background, where reproducibly higher levels of the PipX protein are produced from the marker fusion Φ(C.K1-*pipX*) [22,23]. One-point mutation derivatives are obtained (collectively called CK1X*Y, where the asterisk symbolises each of the PipX mutations), and they are used as recipients for the inactivation allele *glnB*::C.S3. Alternatively, if CK1X*Y strains are not viable (that is, there is no complete segregation of the Φ(C.K1-pipX*) allele), the corresponding mutations are classified as gain-of-function.

We next introduced R70A and L80Q variants in the appropriate plasmids and strains and tested them for PipX toxicity alongside a wild-type control and other mutations of interest. The latter included two substitutions at the TLD/KOW that were not previously analysed for toxicity: H9A, known to impair Y2H signals with both PII and NtcA, and Y16A, which only impaired Y2H signals with PII [17].

As shown in Figure 2B, the *glnB*::C.S3 allele was segregated in strains CK1X^H9A^Y and CK1X^L80Q^Y (CK1X^H9A^Y-B and CK1X^L80Q^Y-B in Table 1) but not in CK1X^Y16A^Y, CK1X^R70Q^Y, or the wild-type control, thus indicating that H9A and L80Q, but not Y16A or R70A, suppressed toxicity.

Since neither R69A nor R70A suppress toxicity, it appears that the R-rich patch is not directly involved in toxicity, although we cannot exclude the possibility of functional redundancy between R70 and its neighbour R69, which also does not suppress toxicity. On the other hand, suppression of toxicity by the L80Q variant supported the importance of the flexed conformation for the toxic function of PipX.

The location of PipX structure and toxicity phenotypes conferred by these and all previously studied mutations are illustrated in Figure 2C. Note that residues to be mutated were chosen in the context of studies on PipX interactions with PII, NtcA, or, to a lesser extent, PlmA, and thus their distribution along the PipX sequence is non-random.

### 3.2. Most but Not All PipX Point Mutations Alter Toxicity and NtcA Coactivation in a Similar Manner

Because toxicity is also triggered by PipX overexpression [23,37,39], a very simplistic explanation for the loss- or gain-of-function mutations would be that they correlate with, respectively, lower or higher levels of PipX. However, previous studies concerning the two spontaneous mutations suppressing PipX toxicity (R54C and L65Q; [22]) and the two gain-of-function mutations contradicted the idea, since that correlation stands only for L65Q and, remarkably, the gain-of-function proteins PipX^E4A^ or PipX^Y32A^ are detected at lower levels than PipX in the corresponding CS3XY derivative strains [23]. The implication is that toxicity must depend on the increased binding of these gain-of-function proteins to additional partner(s).

The possibility that PipX toxicity was due to the upregulation of NtcA target genes prompted studies on the effect of mutations altering toxicity on the co-activation of NtcA-dependent genes [22,23,28]. Altogether, there is a good but incomplete correlation between the effects of mutations in NtcA coactivation and toxicity. All three tested mutations that were wild-type for PipX toxicity were also wild-type for NtcA coactivation (R69A, Q82A, and Q86A), while the two mutations that increased toxicity (E4A and Y32A) and most mutations that decreased PipX toxicity (Y6A, Q34A, R35A, F38A, R54C, and L65Q) were, respectively, gain-of-function and loss-of-function for NtcA coactivation. However, three loss-of-function mutations for toxicity (F12A, D23A, and L36A) did not decrease NtcA coactivation, indicating separation of functions. In line with this, PII also counteracted PipX toxicity under conditions in which NtcA is known to be mainly inactive, as is the case in cultures with ammonium as a nitrogen source [32]. Therefore, it appears that upregulation of NtcA gene targets might contribute to but would not be the only factor involved in PipX toxicity.

### 3.3. Not All Loss-of-Function Mutations for Toxicity Decrease PipX Levels in S. elongatus

We wondered to what extent loss-of-function for toxicity was caused by mutations not impairing PipX levels in *S. elongatus*, since they would inform us of the determinants and molecular mechanisms involved. Next, we obtained CK1X*Y strains corresponding to each of the loss-of-function or neutral mutations available and subsequently performed western blots to compare PipX levels. It is worth noting that the effect on PipX* levels of mutations E4A and Y32A could not be tested in parallel since they are lethal in strain CK1XY. However, they were previously shown to decrease PipX* levels in strain CS3XY [23].

**Figure 3:**
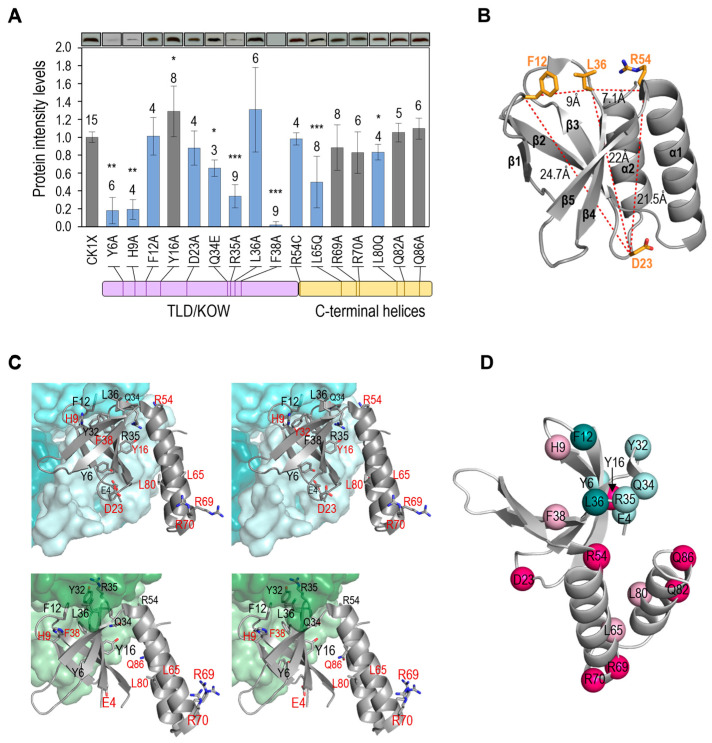
Effects of PipX point mutations on protein levels and location of residues in complexes. (**A**) Top, representative illustrations of PipX bands from Western blots of *S. elongatus* derivatives Main panel: quantification (means and SD) of PipX band intensities for the indicated number of biological replicates (above the bars) normalised to the intensity in the same blot of endogenous PlmA. The Wilcoxon rank sum test with Holm–Bonferroni correction of the mutants versus the control strain (CK1XY) produced *p*-values <0.05 (∗), <0.01 (**), or <0.001 (***). The colours of the bars follow the colour code in Figure 2C for toxicity. Bottom: a schematic representation of the PipX polypeptide indicates the position of mutations. (**B**) Localization in the PipX structure (chain E taken from PDB 2XG8; secondary structure elements are labelled) of residues inferred to provide determinants for toxicity, with side chains shown in stick representation and inter-residue distances as dashed red lines. C, O, and N atoms are coloured orange, red, and blue, respectively. (**C**) Stereoviews of PipX-PII (top) or PipX-NtcA (bottom) zooming on PipX (in cartoon representation) and the region of PII or NtcA (in surface representation) that accommodates PipX. Side chains of PipX residues are shown in stick representation (O and N atoms in red and blue, respectively) and in black or red coloring, depending on whether they do or do not make direct interactions with PII or NtcA. (**D**) The same residues are represented as spheres on the isolated PipX structure (from PDB:2XG8 chain E) and residues interacting or not with PII in cyan or pink, respectively. Pale and dark tones correspond to low or normal protein levels, respectively.

As shown in Figure 3A, significantly lower levels of PipX* were found amongst most of the strains carrying loss-of-function mutations for toxicity (Y6A, H9A, Q34E, R35A, F38A, L65Q, and L80Q), while not amongst any of the strains classified as wild-type for toxicity (carrying substitutions Y16A, R69A, R70Q, Q82A, or Q86A). On the other hand, the variant Y16A, while not altering toxicity, conferred slightly higher levels of PipX*.

Four mutations decreased toxicity without significantly influencing the levels of PipX (F12A, D23A, L36A, and R54C). Three of them replace residues of the TLD/KOW domain (F12, D23, L36), mapping at loops on opposite edges of the β-sheet (loops β1-β2 and β2-β3, respectively) or at the beginning of β4, on the same edge of the sheet as F12 (Figure 3B). The fourth (R54C) is at the beginning of the C-terminal domain and in close proximity to L36. Mutations F12A, L36A, and R54C impair the interaction of PipX with at least NtcA, and although mutations F12A and L36A did not impair NtcA coactivation of reporter genes [23], we cannot exclude the possibility that suppression of PipX toxicity by these mutations might derive from a negative effect on the coactivation of other NtcA-dependent genes. This may be the case of R54C, specifically altering yeast-two hybrid interaction with NtcA and impairing coactivation [22]. However, residue D23 makes no direct contact with PII or NtcA (see PDB files 2XG8 and 2XKO), and thus the same mechanism for suppression of PipX toxicity would not apply here.

In addition, some, if not all, residues identified by mutations decreasing both toxicity and PipX levels could also participate in toxic interactions. Unfortunately, the distinction between direct and indirect involvement for those residues would require protein-protein interaction assays with the corresponding PipX partner. However, the information gained here concerning toxicity and protein levels should help in the search for that putative protein by performing protein-protein interaction assays between candidate proteins and PipX mutant derivatives.

### 3.4. Most Mutations Impairing Binding to PII and NtcA Reduce PipX Levels in S. elongatus

The finding that several mutations targeting residues that bind to PII or NtcA, including the two known gain-of-function mutations (E4A and Y32A), impaired PipX levels in *S. elongatus* suggested to us that the stability of PipX in vivo may depend on its ability to form complexes with its more abundant partners, particularly with PII. Interestingly, proteins PipX^E4A^, PipX^Y6A^, or PipX^Y32A^, detected at low levels in *S. elongatus* ([23]; Figure 3A), were not particularly unstable when expressed in *E. coli* [17], suggesting that PipX may be more susceptible to proteases in cyanobacteria.

To further explore the idea that the stability of PipX in vivo may depend on its ability to form complexes with PII or NtcA, we determined PipX-PII and PipX-NtcA contacts using LigPlot^+^, using PDBs 2XG8 and 2XKO complexes for PipX chains E and C (Figure 3C).

The most drastic effects on PipX* protein levels were produced by the substitution F38A, targeting an invariant residue at β4 that has a key structural role in filling the hydrophobic nucleus to stabilise the curved conformation of the TLD/KOW domain [17,23]. Thus, F38A misfolding effects are independent of PipX interactions. The three substitutions in the TLD/KOW domain that decreased PipX levels (Y6A, Q34A, and R35A) involve residues with extensive interactions with PII and NtcA, while the substitutions that do not decrease PipX levels (Y16A and D23A) target residues having no direct interaction with PII or NtcA.

Two mutations suggested that binding to PII may be more critical for PipX levels than binding to NtcA: E4A, which specifically impairs binding to PII and decreases PipX levels, and R54C, which specifically impairs binding to NtcA and does not decrease PipX levels. Given that under standard culture conditions, PipX monomers are an order of magnitude more abundant than NtcA monomers, while PII trimers are in a 40-fold excess over NtcA dimers [48], a role as PipX chaperon would make physiological sense in the case of PII but not so much for NtcA.

While the results available so far support the notion that the stability of PipX in vivo was affected by its ability to form complexes, particularly with PII (summarised in Figure 3D), two mutations were apparently at odds with this idea. F12A and L36A target residues at the PII and NtcA interaction surfaces of the TLD/KOW domain and abolish Y2H interaction signals with PII and with NtcA [17] but do not reduce PipX* levels or impair NtcA co-activation [23]. Thus, it appears that cellular factors in *S. elongatus* may specifically ameliorate the impact of these substitutions on interactions with PII and NtcA or that the mutations stabilise PipX, perhaps by increasing its affinity for alternative partners.

The L65Q and L80Q variants, which confer lower levels of PipX*, represent residues outside the TDL/KOW domain. Since L65 and L80 are involved in the maintenance of the flexed conformation, which is the conformation adopted by PipX when not bound to its targets [47] it is tempting to propose that the flexed form contributes to PipX stability.

Altogether, the results of mutational analyses in *S. elongatus* suggest that complex formation regulates the stability of PipX, raising questions about the physiological significance and molecular details involved.

### 3.5. PipX Levels Are Down Regulated by Switching from Dark to Light Conditions

No significant changes in PipX levels of *S. elongatus* cultures subjected to various conditions of nitrogen, temperature, or light regimes in different studies have been reported [23,40,48]. However, the above-discussed results suggest that transient decreases in PipX levels may occur in response to environmental changes that disrupt PipX-PII complexes.

Disruption of PipX-PII complexes can be triggered by transferring to light cultures that have been previously maintained in darkness, an environmental change known to increase the intracellular ATP/ADP ratio in *S. elongatus* [32,49].

**Figure 4:**
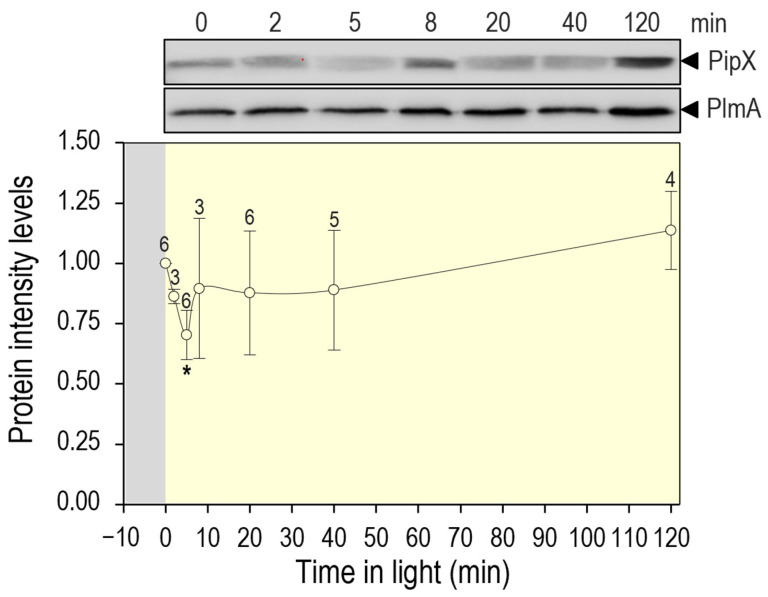
PipX levels after the transition from darkness to light. Representative immunodetection pictures of PipX and PlmA detected in *S. elongatus* and relative PipX levels, normalised by the PlmA signal and referred to as point 0, Means and error bars (SD) from the number of biological replicates (two independent experiments) are indicated above the points. The Wilcoxon rank sum test with Holm–Bonferroni correction versus the 120-min point produced *p*-values < 0.05 (*).

To provide independent experimental support for the positive role of PII binding in the stability of PipX, we next determined PipX levels in *S. elongatus* cultures subjected to darkness and at different times after being illuminated. Cultures were subjected to a 12 h period of darkness before being transferred to light, and timepoints were taken at different intervals up to 120 min. As shown in Figure 4, a 30% decrease in PipX levels was already observed 5 min after the light switch, followed by a slow recovery that was complete at the end of the experiment. Therefore, the results confirm that disrupting PipX-PII complexes results in a transient decrease in PipX levels.

### 3.6. Regulatory Complexities of the PipX-PII Interaction Network, the Dual Role of PII

Sequestration into PipX-PII complexes has been regarded as a molecular mechanism for PII to negatively control NtcA transcriptional activity [12,17] and interfere with the formation of the different PipX complexes in response to intracellular signals of carbon/nitrogen or energy status. However, we show here that PII-PipX complex formation also provides a mechanism to maintain a relatively large pool of PipX, implying that PII, acting as a PipX chaperon, is also an activator of PipX.

Before we reported the identification of PipX [11,12], a positive role as a regulator of NtcA-activated genes was suggested for *S. elongatus* PII [50]. However, we subsequently found that the inactivation of PII requires the obtention of suppressor mutations that often map at *pipX* [21,24], and thus PipX deficiency provided a straightforward explanation for the impaired NtcA activity in a *glnB* null background. The present study provides a molecular mechanism for the positive regulatory role of PII on NtcA activity and other PipX-controlled processes. PII would thus have dual regulatory roles over different PipX complexes: interference, favoured by the great excess of PII over other PipX partners [32,39], and stimulation of the formation of those other PipX complexes, favoured by the PipX-storage role of PII.

The positive role of PII on PipX levels raises questions about the extent to which the levels of other PII-binding proteins, particularly small regulatory ones, are modulated by complex formation with PII. Small proteins appear to play critical roles in key cyanobacterial processes such as photosynthesis or regulation of the carbon-to-nitrogen balance [51,52,53,54,55,56,57,58,59], with paradigmatic examples such as the inhibition by nitrogen abundance of glutamine synthetase, executed by two small proteins instead of by covalent modification, the main mechanism in proteobacteria [60,61,62,63]. Recent work in *Synecocystis* sp. PCC 6803 [64,65,66] has shown that PII binds other small proteins, collectively called Pirs (PII-interacting regulators). Two of them, PirA and PirC/CfrA, were highly enriched in the PII-target searches [64] and have been shown to be involved in the regulation of the carbon-to-nitrogen balance. PirA antagonises the PII-dependent activation of NAGK, the key enzyme in arginine biosynthesis, and can be considered a PII-sequestrator, while PirC allosterically inhibits the glycolytic enzyme 2,3-phosphoglycerate-independent phosphoglycerate mutase (PGAM), and it is thus sequestered by PII, as is the case with PipX. However, the levels of PirA and PirC are highly [64,65,66] upregulated, a common feature shared by small regulatory proteins. In contrast, PipX is so far the only small cyanobacterial protein whose levels appear to be transiently downregulated in response to signals that trigger disruption of PII-PipX complexes.

### 3.7. PipX Complexes and Toxicity

The interaction between PipX and EngA, an essential protein involved in translation that is modulated in response to GTP and GDP levels [67,68], is so far the best candidate for PipX toxicity. GTP/GDP ratios are expected to increase in *S. elongatus* during the transition from darkness to light prior to reinitiating culture growth [69]. In addition, in vitro pull-down assays suggested that the binding of PipX to EngA is decreased by GDP [39]. Thus, immediately after the transfer of cultures from darkness to light, disruption of PipX-PII complexes combined with an increased GTP/GDP ratio would promote partner swapping and the formation of PipX-EngA complexes. This partner swapping of PipX from PII to less abundant targets such as EngA would be followed by a transient decrease in PipX levels, which would allow additional redistribution of PipX in relation to its partners and presumably the separation of “toxic” PipX-EngA complexes that would stimulate translation and growth. Further work would address whether PipX-EngA interactions are disrupted by the mutations identified here as directly involved in PipX toxicity.

## 4. Conclusions

To gain further insights into the regulatory complexities of PipX, we have studied the impact of independent substitutions on PipX toxicity and protein levels in *S. elongatus*. As a result, we have identified four mutations that suppress toxicity without impairing the levels of PipX in *S. elongatus*. Their location suggests that the toxic interaction may involve PipX regions overlapping only partially with the surface binding to PII or NtcA, a scenario incompatible with the formation of ternary complexes with PII or NtcA. The residues identified as determinants of PipX toxicity provide new insights into the phenomenon, and mutations that suppress toxicity without impairing the levels of PipX can now be tested for interactions with PipX-binding partners.

As a result of the mutational analysis presented here, we have been able to infer, and subsequently demonstrate, that PII regulates PipX levels in *S. elongatus*. This introduces a previously ignored additional level of control in the context of the PipX interaction network.

## Figures and Tables

**Table 1:** Strains.

Strain	Genotype or Relevant Characteristics	Source or Reference
*E. coli* DH5α	F^−^ φ80 *lacZ*ΔM15 Δ(*lacZYA-argF*)U169 *endA1 recA1 hsdR17*(r_K_^−^ m_K_^+^) *deoR phoA thi-1 supE44 gyrA96 relA1* λ^−^	[44]
WT	Wild type *S. elongatus* PCC7942	Pasteur Culture Collection
∆*pipX*	*pipX*::*cat*, Cm^R^	[33]
CK1XY	Φ(C.K1-*pipXpipY*), Km^R^	[12]
CK1X^Y6A^Y	Φ(C.K1-*pipX*^Y6A^*pipY*), Km^R^	[23]
CK1X^H9A^Y	Φ(C.K1-*pipX*^H9A^*pipY*), Km^R^	This work
CK1X^H9A^Y-B	Φ(C.K1-*pipX*^H9A^*pipY*)/*glnB*::CS3, Km^R^ Sm^R^	This work
CK1X^F12A^Y	Φ(C.K1-*pipX*^F12A^*pipY*), Km^R^	[23]
CK1X^Y16A^Y	Φ(C.K1-*pipX*^Y16A^*pipY*), Km^R^	This work
CK1X^D23A^Y	Φ(C.K1-*pipX*^D23A^*pipY*), Km^R^	[23]
CK1X^Q34E^Y	Φ(C.K1-*pipX*^Q34E^*pipY*), Km^R^	[23]
CK1X^R35A^Y	Φ(C.K1-*pipX*^R35A^*pipY*), Km^R^	[23]
CK1X^L36A^Y	Φ(C.K1-*pipX*^L36A^*pipY*), Km^R^	[23]
CK1X^F38A^Y	Φ(C.K1-*pipX*^F38A^*pipY*), Km^R^	[23]
CK1X^R54C^Y	Φ(C.K1-*pipX*^R54C^*pipY*), Km^R^	[22]
CK1X^L65Q^Y	Φ(C.K1-*pipX*^L65Q^*pipY*), Km^R^	[22]
CK1X^R69A^Y	Φ(C.K1-*pipX*^R69A^*pipY*), Km^R^	[23]
CK1X^R70A^Y	Φ(C.K1-*pipX*^R70A^*pipY*), Km^R^	This work
CK1X^L80Q^Y	Φ(C.K1-*pipX*^L80Q^*pipY*), Km^R^	This work
CK1X^L80Q^Y-B	Φ(C.K1-*pipX*^L80Q^*pipY*)/*glnB*::CS3, Km^R^ Sm^R^	This work
CK1X^Q82A^Y	Φ(C.K1-*pipX*^Q82A^*pipY*), Km^R^	[23]
CK1X^Q86A^Y	Φ(C.K1-*pipX*^Q86A^*pipY*), Km^R^	[23]

**Table 2:** Plasmids.

Plasmid	Genotype or Relevant Characteristics	Source or Reference
pUAGC126	*pipX* replaced with *cat*, Ap^R^ Cm^R^	[33]
pUAGC701	*CS3(-) into glnB,* Ap^R^ Sm^R^	[21]
pUAGC410	[Φ(C.K1-*pipXpipY*)], Ap^R^ Km^R^	[12]
pUAGC685	[Φ(C.K1-*pipX*^Y6A^*pipY*)], Ap^R^ Km^R^	[23]
pUAGC948	[Φ(C.K1-*pipX*^H9A^*pipY*)], Ap^R^ Km^R^	This work
pUAGC939	[Φ(C.K1-*pipX*^F12A^*pipY*)], Ap^R^ Km^R^	[23]
pUAGC945	[Φ(C.K1-*pipX*^Y16A^*pipY*)], Ap^R^ Km^R^	This wok
pUAGC680	[Φ(C.K1-*pipX*^D23A^*pipY*)], Ap^R^ Km^R^	[23]
pUAGC687	[Φ(C.K1-*pipX*^Q34E^*pipY*)], Ap^R^ Km^R^	[23]
pUAGC688	[Φ(C.K1-*pipX*^R35A^*pipY*)], Ap^R^ Km^R^	[23]
pUAGC976	[Φ(C.K1-*pipX*^L36A^*pipY*)], Ap^R^ Km^R^	[23]
pUAGC940	[Φ(C.K1-*pipX*^F38A^*pipY*)], Ap^R^ Km^R^	[23]
pUAGC681	[Φ(C.K1-*pipX*^R54C^*pipY*)], Ap^R^ Km^R^	[22]
pUAGC682	[Φ(C.K1-*pipX*^L65Q^*pipY*)], Ap^R^ Km^R^	[22]
pUAGC689	[Φ(C.K1-*pipX*^R69A^*pipY*)], Ap^R^ Km^R^	[23]
pUAGC937	[Φ(C.K1-*pipX*^R70A^*pipY*)], Ap^R^ Km^R^	This work
pUAGC618	[Φ(C.K1-*pipX*^L80Q^*pipY*)], Ap^R^ Km^R^	This work
pUAGC619	[Φ(C.K1-*pipX*^Q82A^*pipY*)], Ap^R^ Km^R^	[23]
pUAGC683	[Φ(C.K1-*pipX*^Q86A^*pipY*)], Ap^R^ Km^R^	[23]

Ap, ampicillin; Sm, streptomycin; Cm, chloramphenicol; Km, kanamycin; ^R^, resistance; cat, chloramphenicol acetyltransferase.

## Data Availability

Supporting data are available from the authors if requested.

## Supplementary Materials

The following supporting information can be downloaded at: https://www.mdpi.com/article/10.3390/microorganisms11102379/s1, Table S1: Oligonucleotides, and Table S2: Western blot quantification data.
