# Supplementary material for: The Signal Transduction Protein PII Controls the Levels of the Cyanobacterial Protein PipX
Source: Microorganisms. 2023 Sep 23;11(10):2379. doi: 10.3390/microorganisms11102379 (PMC10609283; doi:10.3390/microorganisms11102379)
Supplement: Supplementary file 1 [file microorganisms-11-02379-s001.zip › Table S1. Oligonucletides.pdf]

Table S1. Oligonucleotides.

Name	Sequence (5'— 3')
GlnB-1F	GGCTTAAGGAGAATTCCCTTGAAGAAG
GlnB-1R	AACTGCAGTCGACGCTGACTTAGATTGCGTCG
PipX-126F	TAAAAACTAGCCGCCCTTGC
PipX-5R	CAGCCCGCAAATCAGCAG
PipX-H9A-1F	CTACCTCAACGCTCCCACCTTCG
PipX-H9A-1R	CGAAGGTGGGAGCGTTGAGGTAG
PipX-Y16A-F	CCCACCTTCGGATTGCTCGCCCAAATCTGCAGC
PipX-Y16A-R	GCTGCAGATTTGGGCGAGCAATCCGAAGGTGGG
PipX-R70A-F	CCGTCTGCGCCAGCTGCGCGCAGATGCCAGTCTGCAGGAATA
PipX-R70A-R	TATTCCTGCAGACTGGCATCTGCGCGCAGCTGGCGCAGACGG
PipX-L80Q-F	GCAGGAATACAACCAGCAGCAGCAAGTCTTCAAAC
PipX-L80Q-R	GTTTGAAGACTTGCTGCTGCTGGTTGTATTCCTGC
